# Bioactive Surface Modification of Hydroxyapatite

**DOI:** 10.1155/2013/626452

**Published:** 2013-06-05

**Authors:** Yasuhiko Abe, Yohei Okazaki, Kyou Hiasa, Keisuke Yasuda, Keisuke Nogami, Wataru Mizumachi, Isao Hirata

**Affiliations:** ^1^Department of Advanced Prosthodontics, Applied Life Sciences, Institute of Biomedical & Health Sciences, Hiroshima University, 1-2-3, Kasumi, Minami-ku, Hiroshima 734-8553, Japan; ^2^Department of Biomaterial Science, Basic Life Sciences, Institute of Biomedical & Health Sciences, Hiroshima University, 1-2-3, Kasumi, Minami-ku, Hiroshima 734-8553, Japan

## Abstract

The purpose of this study was to establish an acid-etching procedure for altering the Ca/P ratio of the nanostructured surface of hydroxyapatite (HAP) by using surface chemical and morphological analyses (XPS, XRD, SEM, surface roughness, and wettability) and to evaluate the *in vitro* response of osteoblast-like cells (MC3T3-E1 cells) to the modified surfaces. This study utilized HAP and HAP treated with 10%, 20%, 30%, 40%, 50%, or 60% phosphoric acid solution for 10 minutes at 25°C, followed by rinsing 3 times with ultrapure water. The 30% phosphoric acid etching process that provided a Ca/P ratio of 1.50, without destruction of the grain boundary of HAP, was selected as a surface-modification procedure. Additionally, HAP treated by the 30% phosphoric acid etching process was stored under dry conditions at 25°C for 12 hours, and the Ca/P ratio approximated to 1.00 accidentally. The initial adhesion, proliferation, and differentiation (alkaline phosphatase (ALP) activity and relative mRNA level for ALP) of MC3T3-E1 cells on the modified surfaces were significantly promoted (*P* < 0.05 and 0.01). These findings show that the 30% phosphoric acid etching process for the nanostructured HAP surface can alter the Ca/P ratio effectively and may accelerate the initial adhesion, proliferation, and differentiation of MC3T3-E1 cells.

## 1. Introduction

 Hydroxyapatite [Ca_10_(PO_4_)_6_(OH)_2_] (HAP) and *β*-tricalcium phosphate [*β*-Ca_3_(PO_4_)_2_] (*β*-TCP) are candidate materials for bone tissue engineering scaffolds because they have bioactive and osteoconductive properties *in vivo* [1]. These materials support the adhesion, proliferation, and differentiation of osteogenesis-related cells such as osteoblasts and mesenchymal stem cells. HAP is bioactive but not bioresorbable and is most thermodynamically stable at a physiological pH (7.4) [2]. In contrast, *β*-TCP has a high biodegradation rate but does not have the desired mechanical properties such as bending strength and fracture toughness [3]. Therefore, the biphasic calcium phosphate (BCP) concept is determined by the optimum balance between the more stable phase of HAP and the more soluble *β*-TCP. *In vivo* bioresorbability of BCP can be controlled by phase composition because the reactivity of BCP increases as the *β*-TCP/HAP ratio increases [4, 5]. Recently, bioactive glasses (BGs) were shown to exhibit especially high bioactivity by releasing dissolution ions such as Ca, P, and Si, which may affect both gene expression and vascularization in osteogenic cells and, subsequently, promote a high rate of bone formation [6]. However, the exact mechanisms underlying the interaction between the ionic dissolution products of such BGs and human cells are not fully understood [1].

Presently, scaffolds fabricated from HAP that exhibit high porosity and pore interconnectivity are used clinically [1, 2, 7, 8]. Thus, it is necessary to design a simple and reproducible approach for enhancing the bioactive surface of HAP by modifying the Ca/P ratio of its nanostructured surface. A bioactive surface with a Ca/P ratio of less than 1 is not suitable for implantation into the body because of its high solubility and acidity [2]; therefore, it had been proposed that the super surface layer of HAP (Ca/P ratio = 1.67) should be modified to mimic that of *β*-TCP (Ca/P ratio = 1.50) [9, 10].

Dorozhkin [11, 12] illustrated the processes of solubilization, the equilibrium between the HAP surface and aqueous solutions, and the interaction between organisms and HAP surfaces very well. According to Dorozhkin, HAP dissolution occurs through 5 steps as follows:

1. Ca_5_(PO_4_)_3_OH_(s)_ + H^+^_(aq)_⇄Ca_5_(PO_4_)_3_(H_2_O)^+^_(s)_
2. 2Ca_5_(PO_4_)_3_(H_2_O)^+^_(s)_⇄3Ca_3_(PO_4_)_2(s)_ + Ca^2+^_(aq)_ + 2H_2_O_(aq)_
3. Ca_3_(PO_4_)_2(s)_ + 2H^+^_(aq)_⇄Ca^2+^_(aq)_ + 2CaHPO_4(s)_
4. CaHPO_4(s)_ + H^+^_(aq)_⇄Ca^2+^_(aq)_ + H_2_PO_4__(aq)_^−^
5. CaHPO_4(s)_⇄Ca^2+^_(aq)_ + HPO_4__(aq)_^2−^

**Figure 1:**
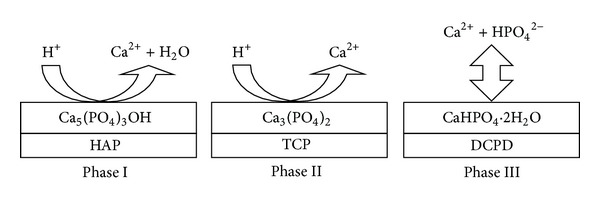
Modified schematic diagram representing the phenomena that occur on the surface of hydroxyapatite (Ca_10_(PO_4_)_6_(OH)_2_: HAP) after implantation, illustrated by Bertazzo et al. [13]. Phase I: beginning of the implant procedure, where the solubilization of the HAP surface starts. Phase II: continuation of the solubilization of the HAP surface (tricalcium phosphate, Ca_3_(PO_4_)_2_: TCP). Phase III: achievement of equilibrium between physiological solutions and the modified surface of HAP (dicalcium phosphate dehydrate (brushite), CaHPO_4_ *·*2H_2_O: DCPD).

These 5 steps show that, throughout the process of HAP dissolution, the composition of the surface changes to Ca_3_(PO_4_)_2_ (tricalcium phosphate, TCP) and CaHPO_4_ (dicalcium phosphate dehydrate, DCPD). Then, Bertazzo et al. [13] proposed 3 initial phases that occur before equilibrium is established between the HAP surface and biological fluids (Figure 1). With time, equilibrium is reached between the modified HAP surface and biological fluids, hence forming the compound DCPD on this surface (phase III in Figure 1). Following the achievement of equilibrium on the surface, there are steps that lead to the integration of the HAP surface with the tissue, such as protein adsorption and cell adhesion.

We put forth the hypothesis that the acid-etching procedure, altering the Ca/P ratio of the HAP surface directly by phosphoric acid, that has been described in this study can cause the bioactive surfaces to mimic the initial phases proposed by Bertazzo et al. [13] because the sintering process for acquiring the desired surface of HAP is complex and unstable [2]. Therefore, the purpose of this study was to establish an acid-etching procedure for altering the Ca/P ratio of the nanostructured surface of HAP by surface chemical and morphological analyses and to evaluate the *in vitro* response of osteoblast-like cells to the modified surfaces.

## 2. Materials and Methods

### 2.1. Establishment of Surface Modification

#### 2.1.1. Sample Preparation

HAP plates (thickness, 2 mm; width, 10 mm; length, 10 mm) (APP-101; Pentax, Tokyo, Japan) were used in this study. HAP plates were treated with 10%, 20%, 30%, 40%, 50%, or 60% phosphoric acid [H_3_PO_4_] (lot no. T1949; Sigma-Aldrich Japan, Tokyo, Japan) solution for 10 minutes at 25°C, followed by rinsing 3 times with ultrapure water (MilliQ water: >18 MΩcm) (HAP—10% PA, HAP—20% PA, HAP—30% PA, HAP—40% PA, HAP—50% PA, and HAP—60% PA).

#### 2.1.2. X-Ray Photoelectron Spectroscopy (XPS) Analysis

 HAP, HAP—10% PA, HAP—20% PA, HAP—30% PA, HAP—40% PA, HAP—50% PA, and HAP—60% PA plates were mounted individually onto stubs with insulating tape. The surfaces of the plates were chemically analyzed using an X-ray photoelectron spectroscopy (XPS) instrument (AXIS-HS; Kratos, Manchester, UK). The measurements were performed *in vacuo* (≤10^−7^ Pa) with Al-K*α* monochromatic X-rays at a source power of 150 W. The AXIS-HS was equipped with an electron flood gun for charge compensation. Wide- and narrow-scan spectra were acquired at pass energies of 80 and 40 eV, respectively. Peak positions were calibrated by referencing a value of 284.6 eV for the peaks corresponding to C–C and C–H in the C 1s spectrum. After smoothing the narrow scans, a straight line (for C 1s, O 1s, Ca 2p, and P 2p) was applied in the quantification. The relative sensitivity factors used to calculate the atomic ratios from the peak area ratios were 0.278 for C 1s, 0.780 for O 1s, 1.833 for Ca 2p, and 0.486 for P 2p. Reproducibility was guaranteed by obtaining 10 measurements per experimental sample. The data were analyzed using one-way ANOVA and Tukey's test for multiple comparisons (*P* < 0.05).

#### 2.1.3. X-Ray Diffraction (XRD) Analysis

 The thin films of the HAP and HAP—30% PA plates were analyzed using an XRD instrument (Ultima IV; Rigaku, Osaka, Japan). Samples were scanned with Cu-K*α* radiation at 40 kV and 50 mA between 10 and 90 (2*θ*) degrees at a step size of 0.04 degrees and a scan rate of 2 degrees/minute. The results were compared to the ICDD standard for HAP (no. 01-072-1243).

### 2.2. Evaluation of Surface Modification

#### 2.2.1. Sample Preparation

A HAP—30% PA plate that was stored under dry conditions at 25°C for 12 hours (HAP—30% PA—12 h) was added as a new sample. The surface characteristics of HAP, HAP—30% PA, and HAP—30% PA—12 h were evaluated by the following methods.

#### 2.2.2. XPS Depth Profiling Analysis

Samples were analyzed using an XPS instrument at incidence angles of 90, 75, 60, 45, 30, and 15 degrees. The measurements were obtained under the above-mentioned conditions. The data were analyzed using one-way ANOVA and Tukey's test for multiple comparisons (*P* < 0.05).

#### 2.2.3. Scanning Electron Microscope (SEM) Observation, Surface Roughness, and Wettability

An SEM (VE-8800; Keyence, Osaka, Japan) was used to observe the surface topography of samples. The surface roughness (Ra) of samples was determined using a confocal laser scanning microscope (VK-8500; Keyence, Osaka, Japan). The Ra value (*μ*m) was defined as the average value of 5 areas (100 *μ*m × 100 *μ*m) with 9 measuring points. The wettability of sample surfaces was measured using a contact angle meter (Slmage mini; Excimer, Kanagawa, Japan) with the sessile drop method and 3.6 *μ*L ultrapure water; Young's equation was used for data analysis. The data were analyzed using one-way ANOVA and Tukey's test for multiple comparisons (*P* < 0.05).

#### 2.2.4. Initial Adhesion and Proliferation of Osteoblast-Like Cells

 Osteoblast-like cells (MC3T3-E1 derivative cell lines of mouse calvaria (RIKEN BioResource Center, Tsukuba, Japan)) were cultured in Dulbecco's Modified Eagle Medium (D-MEM) (lot no. RNBB4045; Sigma-Aldrich Japan, Tokyo, Japan) containing 10% fetal bovine serum (lot nos. F0926, 027K03911; Sigma-Aldrich Japan, Tokyo, Japan) and 1% penicillin-streptomycin (lot nos. P0781, 060M0811; Sigma-Aldrich Japan, Tokyo, Japan) at 37°C in a humidified atmosphere of 5% CO_2_. The medium was changed twice a week.

MC3T3-E1 cells (1 × 10^4^) were seeded onto HAP, HAP—30% PA, and HAP—30% PA—12 h plates sterilized by ultraviolet (UV) irradiation for 4 hours. The cells were incubated at 37°C and 5% CO_2_. Initial cell adhesion was evaluated at 0.5 and 24 hours and cell proliferation at 1, 4, and 7 days. Cells were seeded on tissue culture plates and incubated as controls over each incubation period.

Initial cell adhesion and cell proliferation were analyzed by a Cell Counting Kit-8 assay (Dojindo Laboratory, Kumamoto, Japan), a colorimetric assay that uses a tetrazolium salt, WST-8, to determine viable cell number. After each incubation period, the growth medium in each well was removed by aspiration and replaced with a mixture of WST-8 and D-MEM in a 1 : 10 ratio. After 2 hours of incubation at 37°C and 5% CO_2_, a 110 *μ*L aliquot of the mixture that reacted with viable cells was dispensed in each well (*n* = 5) of a 96-well microplate. The absorbance was read at 450 nm by using a microplate reader (model 550; Bio-Rad Japan, Tokyo, Japan). The data were analyzed using two-way ANOVA and Tukey's test for multiple comparisons (*P* < 0.05).

#### 2.2.5. Cell Differentiation of Osteoblast-Like Cells

 MC3T3-E1 cells (10 × 10^4^) were seeded onto HAP, HAP—30% PA, and HAP—30% PA—12 h plates as described previously. Alkaline phosphatase (ALP) activity was evaluated at 7, 14, and 21 days after obtaining a confluent cell monolayer. Real-time polymerase chain reaction (PCR) analysis of ALP was carried out at 14, 21, and 28 days after obtaining a confluent cell monolayer.

ALP activity was determined by the SensoLyte *p*NPP secreted ALP reporter gene assay (AnaSpec Corporate Headquarters, San Jose, CA), which is optimized to detect placental ALP activity and uses *p*-nitrophenyl phosphate (*p*NPP) as a colorimetric phosphatase substrate. After each incubation period, the growth medium in each well was removed by aspiration, and each well was rinsed twice with 1 mL of lysis buffer. A mixture (1 mL) of 10 mL lysis buffer and 20 *μ*L Triton X-100 was added to each well, the cells were scraped and suspended, and 1,000 *μ*L of the cell suspension was transferred to a new well and incubated at 37°C and 5% CO_2_ for 10 minutes. An aliquot of the cell suspension (300 *μ*L) was then mixed with 300 *μ*L of the *p*NPP reaction mixture in a separate well. After 60 minutes of incubation, 300 *μ*L of stop solution was added to the cell suspension, followed by shaking for 1 minute. A 150 *μ*L aliquot of the mixture was dispensed to each well (*n* = 5) in a 96-well microplate. The absorbance was read at 405 nm by using the microplate reader.

 Total RNA was extracted using an RNeasy Mini Kit (Qiagen, Tokyo, Japan). The total RNA concentration was adjusted to 10 ng/*μ*L by using a NanoDrop 1000 system (Thermo Fisher Scientific, Wilmington, USA). First-strand cDNA was synthesized from total RNA (10 ng/*μ*L) by using a ReverTra Ace qPCR RT Kit (Toyobo, Osaka, Japan). For each gene, a cycle curve experiment was performed, and the optimal number of PCR cycles was selected according to the results. Real-time PCR analysis was performed for ALP and GAPDH (internal control). The sequences of the primers used in this analysis were as follows: forward, 5′-AGGGTGGACTACCTCTTAGGTCT-3′ and reverse, 5′-TGGTCAATCCTGCCTCCTTCC-3′ for ALP amplification; and forward, 5′-GCCAGCCTCGTCCCGTAG-3′ and reverse, 5′-CAAATGGCAGCCCTGGTGAC-3′ for GAPDH amplification. The primers were purchased from Sigma. The data were analyzed using two-way ANOVA and Tukey's test for multiple comparisons (*P* < 0.05).

## 3. Results

### 3.1. Establishment of Surface Modification

#### 3.1.1. XPS Analysis

 The XPS-determined binding energies (eV) of Ca 2p, P 2p, and Δ(Ca 2p, P 2p); atomic concentrations (at.%); and Ca/P ratios for HAP, HAP—10% PA, HAP—20% PA, HAP—30% PA, HAP—40% PA, HAP—50% PA, and HAP—60% PA are shown in Table 1. As for the binding energies of Δ(Ca 2p, P 2p), there were no significant differences among samples (*P* > 0.05). The XPS-determined Ca/P ratio (1.65 ± 0.02) of HAP matched the theoretical value (1.67). The Ca/P ratios for HAP—10% PA, HAP—20% PA, HAP—30% PA, HAP—40% PA, HAP—50% PA, and HAP—60% PA were significantly smaller than that for HAP (*P* < 0.01). The Ca/P ratio (1.50 ± 0.03) of HAP—30% PA was the smallest and matched the theoretical value of TCP (1.50), although its value was not significantly different from that of HAP—20% PA (*P* > 0.05).

#### 3.1.2. XRD Analysis

**Figure 2:**
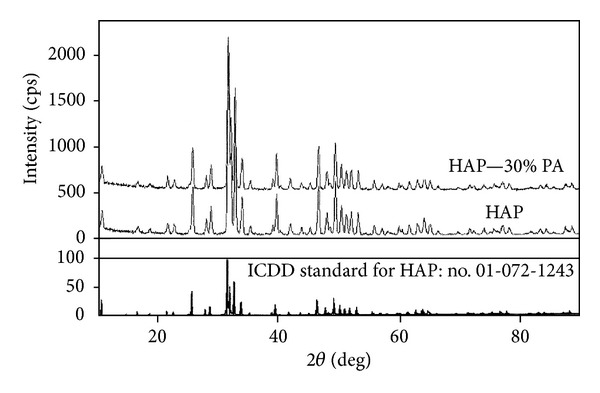
X-ray diffraction (XRD) spectra of HAP and HAP—30% PA thin films referenced to the ICDD standard for HAP (no. 01-072-1243). There was no difference between the XRD spectra of HAP and HAP—30% PA, and the chemical composition of their thin films was assumed to be the same.

 The XRD spectra of HAP and HAP—30% PA thin films, referenced to the ICDD standard for HAP (no. 01-072-1243), are shown in Figure 2. There was no difference between the XRD spectra of HAP and HAP—30% PA, and the chemical composition of their thin films was assumed to be the same.

### 3.2. Evaluation of Surface Modification

#### 3.2.1. XPS Depth Profiling Analysis

The XPS-determined binding energies (eV) of Ca 2p, P 2p, and Δ(Ca 2p, P 2p) and the Ca/P ratios for HAP, HAP—30% PA, and HAP—30% PA—12 h at each incidence angle of the X-ray (90, 75, 60, 45, 30, or 15 degrees) are shown in Table 2. The binding energy of Δ(Ca 2p, P 2p) of HAP—30% PA—12 h (213.91 ± 0.06) was significantly smaller than those of HAP and HAP—30% PA (*P* < 0.05), and there was no significant difference between the binding energies of HAP and HAP—30% PA (*P* > 0.05). As for the Ca/P ratios, there were significant differences among samples (*P* < 0.01), and the values of HAP, HAP—30% PA, and HAP—30% PA—12 h were 1.65 ± 0.04, 1.45 ± 0.01, and 1.09 ± 0.02, respectively. In each sample, changes in the binding energies and the Ca/P ratios were not recognized by the different incidence angles of the X-ray.

#### 3.2.2. SEM, Surface Roughness, and Wettability

**Figure 3:**
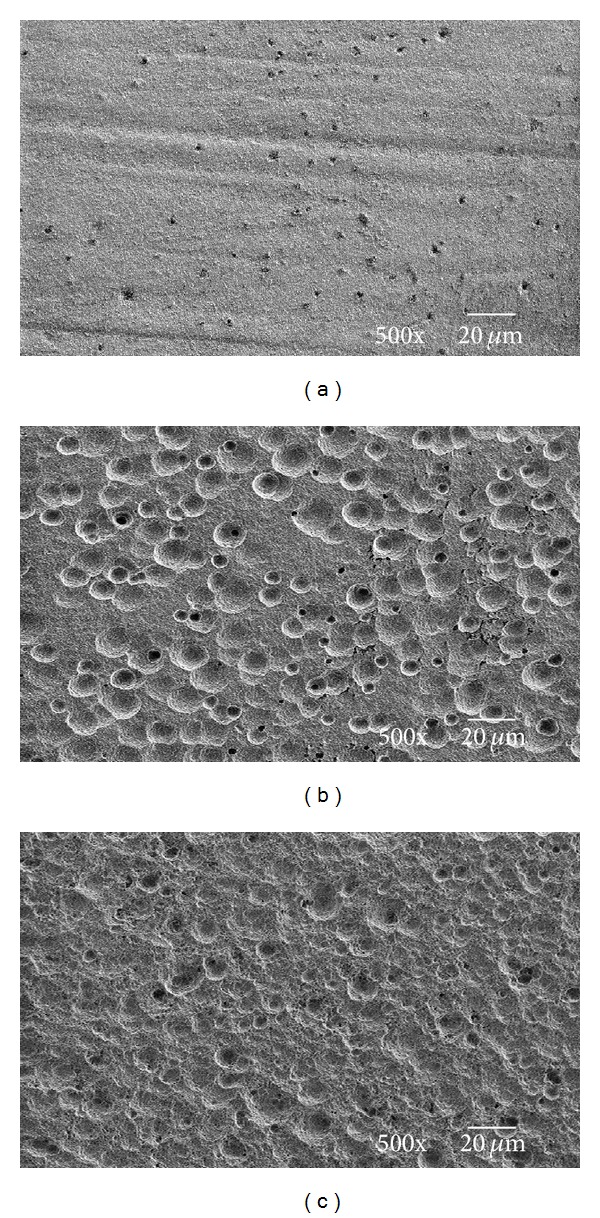
SEM images of (a) HAP, (b) HAP—30% PA, and (c) HAP—30% PA—12 h.

SEM images for HAP, HAP—30% PA, and HAP—30% PA—12 h are shown in Figure 3. The traces with a lack of particles appeared at the surface of HAP—30% PA, and the more scabrous image was observed at the surface of HAP—30% PA—12 h.

 The surface roughness and wettability for HAP, HAP—30% PA, and HAP—30% PA—12 h are shown in Table 3. The surface roughness Ra value of HAP—30% PA—12 h (0.96 ± 0.04 *μ*m) was significantly larger than the Ra values of HAP and HAP—30% PA (0.25 ± 0.06 *μ*m and 0.91 ± 0.05 *μ*m, resp.; *P* < 0.01 and 0.05, resp.). The contact angle of HAP—30% PA—12 h (13.67 ± 3.54 degrees) was significantly smaller than the contact angles of HAP and HAP—30% PA (102.10 ± 2.98 degrees and 55.13 ± 0.35 degrees, resp.; *P* < 0.01).

#### 3.2.3. Initial Cell Adhesion and Proliferation

**Figure 4:**
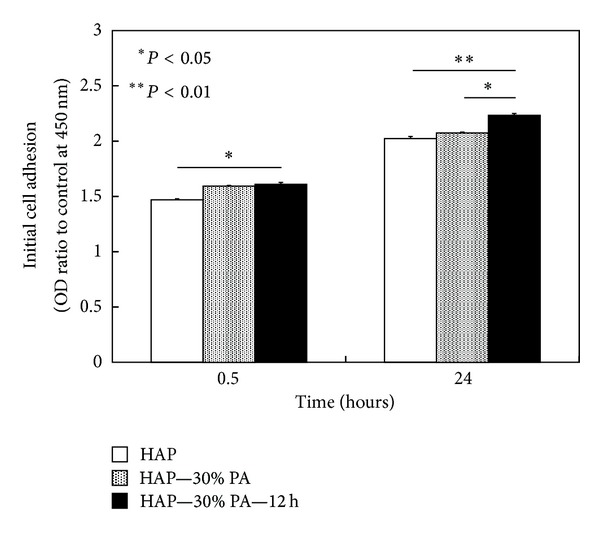
Initial cell adhesion of MC3T3-E1 osteoblast-like cells to HAP, HAP—30% PA, and HAP—30% PA—12 h at 0.5 and 24 hours. At 0.5 hours, the number of cells adherent to HAP—30% PA—12 h was significantly greater than that for HAP (*P* < 0.05). At 24 hours, the number of cells adherent to HAP—30% PA—12 h was significantly greater than those for both HAP (*P* < 0.01) and HAP—30% PA (*P* < 0.05).

The initial cell adhesion of MC3T3-E1 cells to HAP, HAP—30% PA, and HAP—30% PA—12 h at 0.5 and 24 hours is shown in Figure 4. At 0.5 hours, the number of adherent cells for HAP—30% PA—12 h was significantly higher than that for HAP (*P* < 0.05). At 24 hours, the number of adherent cells for HAP—30% PA—12 h was significantly higher than that for HAP (*P* < 0.01) and HAP—30% PA (*P* < 0.05).

**Figure 5:**
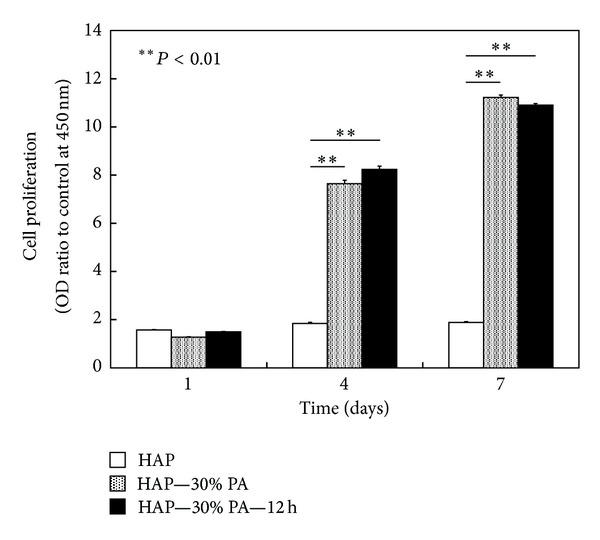
Cell proliferation of MC3T3-E1 osteoblast-like cells on HAP, HAP—30% PA, and HAP—30% PA—12 h at 1, 4, and 7 days. At 4 and 7 days, cell proliferation on HAP—30% PA and HAP—30% PA—12 h was significantly higher than that on HAP (*P* < 0.01); the difference in the cell proliferation on HAP—30% PA and HAP—30% PA—12 h was not statistically different (*P* > 0.05).

The proliferation of MC3T3-E1 cells on HAP, HAP—30% PA, and HAP—30% PA—12 h at 1, 4, and 7 days is shown in Figure 5. At 4 and 7 days, cell proliferation on both HAP—30% PA and HAP—30% PA—12 h was significantly higher than that on HAP (*P* < 0.01); the difference between the growth on HAP—30% PA and HAP—30% PA—12 h was not statistically significant (*P* > 0.05).

#### 3.2.4. Cell Differentiation

**Figure 6:**
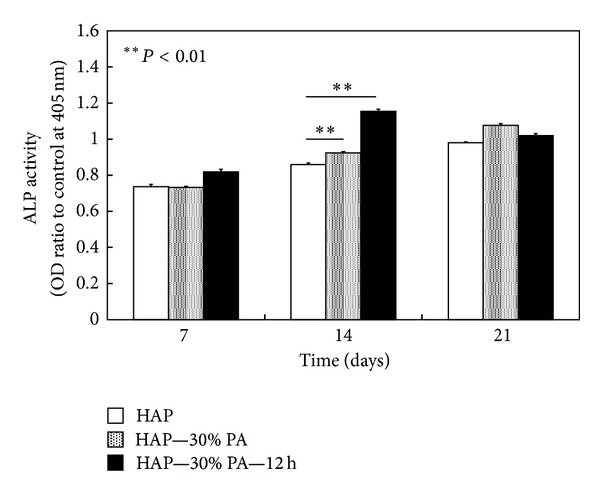
ALP activities of MC3T3-E1 osteoblast-like cells on HAP, HAP—30% PA, and HAP—30% PA—12 h at 7, 14, and 21 days after obtaining a confluent cell monolayer. At 14 days, the ALP activities of the cells on HAP—30% PA and HAP—30% PA—12 h were significantly higher than that on HAP (*P* < 0.01). The ALP activity at 14 days for HAP—30% PA—12 h was higher than that for HAP—30% PA, but the ALP activity at 21 days was lower than that for HAP—30% PA. Although the ALP activities for HAP and HAP—30% PA gradually increased from 7 to 21 days, the ALP activity for HAP—30% PA—12 h reached its peak at 14 days.

 The ALP activity of MC3T3-E1 cells on HAP, HAP—30% PA, and HAP—30% PA—12 h at 7, 14, and 21 days after obtaining a confluent cell monolayer is shown in Figure 6. At 14 days, the ALP activity of the cells on HAP—30% PA and HAP—30% PA—12 h was significantly higher than that on HAP (*P* < 0.01). The ALP activity at 14 days on HAP—30% PA—12 h was higher than that on HAP—30% PA, but the ALP activity at 21 days was lower than that on HAP—30% PA. Although the ALP activities on HAP and HAP—30% PA gradually increased from 7 to 21 days, the ALP activity on HAP—30% PA—12 h reached its peak at 14 days.

**Figure 7:**
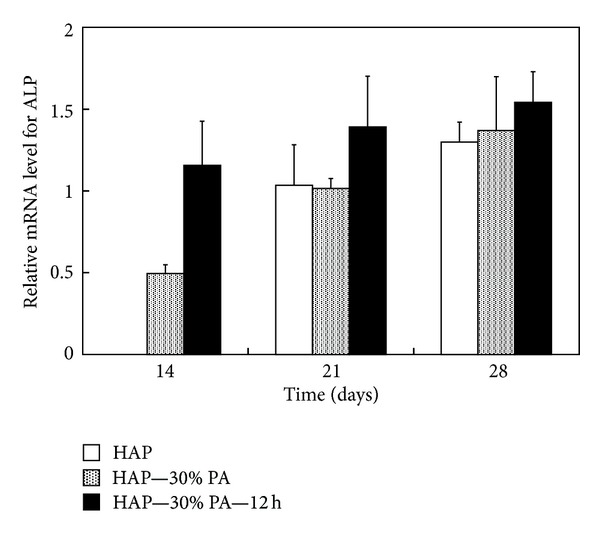
Relative mRNA levels for ALP in MC3T3-E1 osteoblast-like cells on HAP, HAP—30% PA, and HAP—30% PA—12 h at 14, 21, and 28 days after obtaining a confluent cell monolayer. At 14 days, there was no mRNA expression on HAP, but the expression on HAP—30% PA—12 h and HAP—30% PA was estimated in sequence. At 21 days, the expression of ALP on HAP was estimated, and the expression on HAP, HAP—30% PA, and HAP—30% PA—12 h was increased at 28 days. There were no statistically significant differences among expressions at any time (*P* > 0.05).

 Relative mRNA levels for ALP in MC3T3-E1 cells on HAP, HAP—30% PA, and HAP—30% PA—12 h at 14, 21, and 28 days after obtaining a confluent cell monolayer are shown in Figure 7. At 14 days, there was no mRNA expression on HAP, but the expression on HAP—30% PA—12 h and HAP—30% PA was estimated in sequence. The expression on HAP was estimated at 21 days, and the expression on HAP, HAP—30% PA, and HAP—30% PA—12 h was increased at 28 days. No statistically significant differences were observed in the expression at each time point (*P* > 0.05). 

## 4. Discussion

### 4.1. Establishment of Surface Modification

 Modification to the HAP surface by using etching with 10%, 20%, 30%, 40%, 50%, and 60% phosphoric acid altered the Ca/P ratio from 1.50 to 1.58. The Ca/P ratio of HAP—30% PA was the smallest and matched the theoretical value of TCP (1.50). However, there were no significant differences among the binding energies of Δ(Ca 2p, P 2p) for all samples (*P* > 0.05). Since the 30% phosphoric acid etching process did not break the grain boundary of HAP and effectively brought the Ca/P ratio to 1.50, this process was selected as a surface-modification procedure.

 XRD analysis showed that the chemical composition of the thin films (a level of 100 nm) for HAP and HAP—30% PA was assumed to be the same. This finding does not illustrate that the changed Ca/P ratio leads to alteration of the chemical composition of the surface. Thus, the nanostructured surface of HAP—30% PA has not yet been confirmed as being modified to TCP, although the Ca/P ratio was 1.50.

### 4.2. Evaluation of Surface Modification

The Ca/P ratio of HAP—30% PA—12 h was approximated to the Ca/P ratio of DCPD (1.00), but this finding was obtained accidentally. The XPS depth profiling analyses for HAP, HAP—30% PA, and HAP—30% PA—12 h were performed to evaluate the chemical compositions of their nanostructured surfaces. The binding energy of Δ(Ca 2p, P 2p) of HAP—30% PA—12 h was significantly smaller than that of HAP and HAP—30% PA (*P* < 0.05). The Ca/P ratio of HAP—30% PA—12 h (1.09) was significantly smaller than that of HAP—30% PA (1.50) (*P* < 0.01). The SEM results showed a greater degree of scabrousness at the surface of HAP—30% PA—12 h than HAP—30% PA. 

 The reasons for reduction of the Ca/P ratio of the modified surface were (i) Ca defect and (ii) P abundance. P abundance could not be considered because the surface treated with 30% phosphoric acid solution was rinsed with ultrapure water, and the expansion of the break of the grain boundary of HAP was not observed in the SEM image of HAP—30% PA—12 h. The 30% phosphoric acid etching process promotes decalcification of the surface, resulting in the reduction of the Ca/P ratio by Ca defects. As for the reduction of the Ca/P ratio of HAP—30% PA—12 h to 1.00, it is believed that the Ca in the outer layer of the nanostructured surface is supplied to the deep layer for 12 hours to equilibrate the amorphous calcium phosphate.

 Reduction in the Ca/P ratio leads to high solubility of the surface. Additionally, surface modification induces the rougher and wetter surface of HAP. Generally, a contact angle of more than 65 degrees indicates a hydrophobic surface, and a contact angle of less than 65 degrees indicates a hydrophilic surface. Since the contact angle of HAP (102.10 degrees) is greater than 65 degrees, the hydrophobic surface becomes more intense with an increase in surface roughness. However, in this study, the contact angle grew smaller with an increase in surface roughness, resulting in a super hydrophilic surface. These findings demonstrate that the surface might have been modified both morphometrically and chemically.

We expected that the *in vitro* responses of osteoblast-like cells to HAP—30% PA and HAP—30% PA—12 h would be better than those to HAP since the high biodegradation rate of TCP should improve early cell attachment [14, 15]. During initial cell adhesion, HAP—30% PA and HAP—30% PA—12 h were superior to HAP. Cell proliferation on HAP—30% PA and HAP—30% PA—12 h was 4 times higher at 4 days and 6 times higher at 7 days than that on HAP; these differences were statistically significant (*P* < 0.01 for both). MC3T3-E1 cells reached a proliferation peak around day 7 on all substrates, and the cell differentiation process is reflected by ALP activity [16, 17]. The ALP activities at 14 days on HAP—30% PA and HAP—30% PA—12 h were significantly higher than that on HAP (*P* < 0.01) and, in particular, the ALP activity on HAP—30% PA—12 h reached its peak (at 14 days) faster than that on HAP and HAP—30% PA. Moreover, at 14 days, the mRNA expression on HAP—30% PA—12 h and HAP—30% PA was estimated in sequence, but there was no expression on HAP. Intracellular signal transduction during the initial adhesion stage might play a crucial role in cell differentiation and mineralization on calcium phosphate-related biomaterials [14]. Therefore, these findings demonstrate the usefulness of HAP—30% PA and HAP—30% PA—12 h by the 30% phosphoric acid etching process to effectively alter the Ca/P ratio. The hypothesis suggested in this study is supported by our evidence in acquiring bioactive surfaces, but the surface treated with 30% phosphoric acid solution has not yet been confirmed to be modified to TCP or DCPD in the initial phases proposed by Bertazzo et al. [13].

Furthermore, we will investigate the response of osteoclasts to HAP—30% PA and HAP—30% PA—12 h because osteoclasts regulating bone metabolism are coupled with osteoblasts in a direct or indirect manner *in vivo* and involve bone resorption and calcium release.

## 5. Conclusions

 In this study, we established the acid-etching procedure to alter the Ca/P ratio of the nanostructured surface of HAP by surface chemical and morphological analyses and evaluated the *in vitro* response of MC3T3-E1 osteoblast-like cells to the modified surfaces. The 30% phosphoric acid etching process for obtaining a Ca/P ratio of 1.50, without destruction of the grain boundary of HAP, was selected as a surface-modification procedure. Additionally, HAP was treated with 30% phosphoric acid solution and stored under dry conditions at 25°C for 12 hours, and the Ca/P ratio approximated to 1.00 accidentally. The acid-etching procedure for HAP significantly promoted the initial adhesion, proliferation, and differentiation of MC3T3-E1 cells. In future studies, the Ca/P ratio of HAP scaffolds used clinically will be modified, and the usefulness of the scaffolds will be evaluated by *in vivo* studies.

## Figures and Tables

**Table 1:** XPS-determined binding energies (eV) of Ca 2p, P 2p, and Δ(Ca 2p, P 2p); atomic concentrations (at. %); and Ca/P ratios for hydroxyapatite (HAP) and HAP treated with 10%, 20%, 30%, 40%, 50%, or 60% phosphoric acid (HAP—10% PA, HAP—20% PA, HAP—30% PA, HAP—40% PA, HAP—50% PA, and HAP—60% PA).

Sample		Binding energy (eV)			At. %				Ca/P
		Ca 2p	P 2p	Δ(Ca 2p, P 2p)	C 1s	O 1s	Ca 2p	P 2p	
HAP	Mean	346.51	132.45	214.06^a^	26.81	47.74	15.84	9.61	1.65
	SD	0.03	0.05	0.05	2.02	1.35	0.50	0.31	0.02
HAP—10% PA	Mean	346.87	132.80	214.07^a^	11.04	58.00	18.80	12.17	1.54^c^
	SD	0.05	0.05	0.05	1.06	0.58	0.34	0.18	0.01
HAP—20% PA	Mean	346.77	132.72	214.05^a^	15.70	54.75	17.77	11.78	1.51^b^
	SD	0.09	0.09	0.05	6.12	3.76	1.52	0.84	0.02
HAP—30% PA	Mean	346.61	132.54	214.07^a^	25.80	49.32	14.92	9.96	1.50^b^
	SD	0.06	0.07	0.05	1.09	1.36	0.58	0.54	0.03
HAP—40% PA	Mean	346.85	132.79	214.06^a^	9.94	57.77	19.66	12.63	1.56^c,d^
	SD	0.05	0.07	0.05	0.83	0.61	0.18	0.11	0.02
HAP—50% PA	Mean	346.91	132.88	214.03^a^	11.10	57.48	19.19	12.24	1.57^c,d^
	SD	0.07	0.08	0.05	1.80	1.10	0.56	0.25	0.04
HAP—60% PA	Mean	346.73	132.68	214.05^a^	10.93	57.78	19.18	12.11	1.58^d^
	SD	0.05	0.04	0.05	0.32	0.26	0.12	0.12	0.02

The presence of the same superscript letter for the values indicates that there were no significant differences among the samples (*P* > 0.05).

**Table 2:** XPS-determined binding energies (eV) of Ca 2p, P 2p, and Δ(Ca 2p, P 2p), and Ca/P ratios for hydroxyapatite (HAP), HAP treated with 30% phosphoric acid (HAP—30% PA), and HAP—30% PA in storage for 12 hours (HAP—30% PA—12 h) at each incidence angle of the X-ray (90, 75, 60, 45, 30, or 15 degrees).

Sample	Angle of incidence (degrees)	Binding energy (eV)			Ca/P
		Ca 2p	P 2p	Δ(Ca 2p, P 2p)	
HAP	90	346.60	132.55	214.05	1.65
	75	346.70	132.60	214.10	1.59
	60	346.60	132.50	214.10	1.63
	45	346.65	132.55	214.10	1.66
	30	346.60	132.50	214.10	1.69
	15	346.60	132.55	214.05	1.68
	Mean	346.63	132.54	214.08^a^	1.65^b^
	SD	0.04	0.04	0.03	0.04

HAP—30% PA	90	346.80	132.75	214.05	1.45
	75	346.80	132.75	214.05	1.44
	60	346.85	132.75	214.10	1.45
	45	346.85	132.80	214.05	1.44
	30	346.90	132.95	213.95	1.46
	15	346.90	132.65	214.25	1.45
	Mean	346.85	132.78	214.08^a^	1.45^b^
	SD	0.04	0.10	0.10	0.01

HAP—30% PA—12 h	90	347.30	133.40	213.90	1.11
	75	347.25	133.40	213.85	1.10
	60	347.30	133.45	213.85	1.10
	45	347.30	133.40	213.90	1.09
	30	347.30	133.35	213.95	1.11
	15	347.30	133.30	214.00	1.06
	Mean	347.29	133.38	213.91	1.09^b^
	SD	0.02	0.05	0.06	0.02

^a^ *P* > 0.05 indicates no significant difference between the samples.

^b^ *P* < 0.01 indicates significant differences among the samples.

**Table 3:** Surface roughness and wettability for HAP, HAP—30% PA, and HAP—30% PA—12 h.

Sample		Surface roughness	Wettability
		Ra (*μ*m)	Contact angle (degrees)
HAP	Mean	0.25	102.10^c^
	SD	0.06	2.98
HAP—30% PA	Mean	0.91^a^	55.13^c^
	SD	0.05	0.35
HAP—30% PA—12 h	Mean	0.96^a,b^	13.67^c^
	SD	0.04	3.54

Surface roughness: ^a^ *P* < 0.01 indicates significant difference from HAP. ^b^ *P* < 0.05 indicates significant difference from HAP—30% PA.

Wettability: ^c^ *P* < 0.01 indicates significant differences among the samples.
